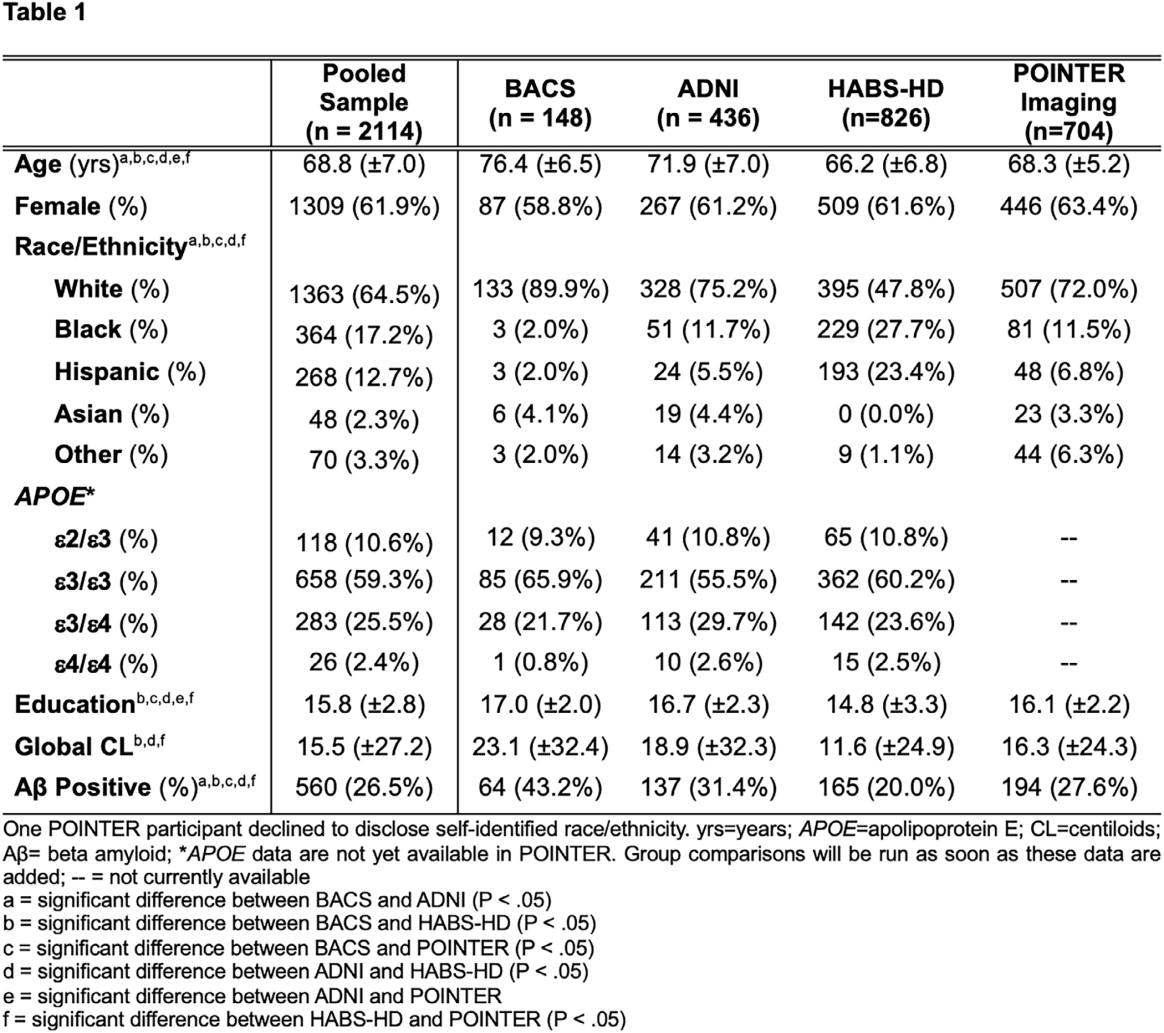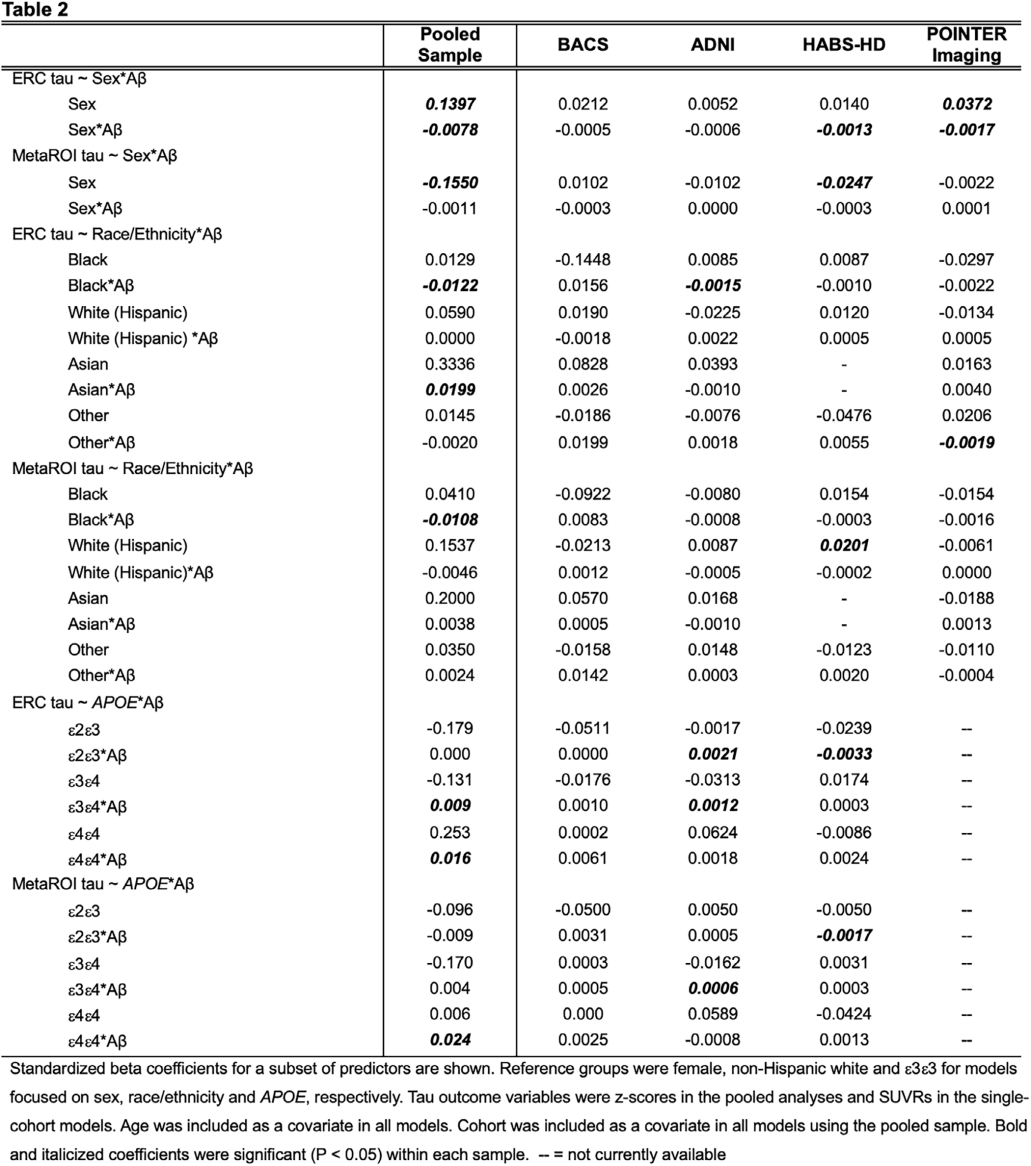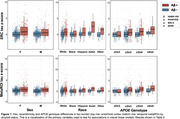# Generalizability of demographic and genetic variable interactions with amyloid driving tau burden in a heterogeneous group of cognitively normal older adults: A multi‐cohort study

**DOI:** 10.1002/alz.092099

**Published:** 2025-01-09

**Authors:** Theresa M. Harrison, Tyler J. Ward, Trevor Chadwick, Jacinda Taggett, Suzanne L. Baker, Meredith N Braskie, Susan M. Landau, William J. Jagust, the U.S. POINTER Study Group, the Alzheimer's Disease Neuroimaging Initiative

**Affiliations:** ^1^ University of California, Berkeley, Berkeley, CA USA; ^2^ Lawrence Berkeley National Laboratory, Berkeley, CA USA; ^3^ Stevens Neuroimaging and Informatics Institute, Los Angeles, CA USA

## Abstract

**Background:**

Efforts to increase heterogeneity in cohorts of cognitively normal older adults with Alzheimer’s disease (AD) neuroimaging biomarkers have resulted in large datasets with differing characteristics. It is unclear whether associations between AD biomarkers and key demographic and genetic factors are generalizable across heterogeneous cohorts.

**Method:**

PET and MR scans from cognitively normal older adults aged >55 in the Berkeley Aging Cohort Study (BACS), ADNI, the Health and Aging Brain Study‐Health Disparities (HABS‐HD) and POINTER Imaging were processed using harmonized pipelines. Global beta‐amyloid (Aβ) burden was quantified in centiloids (CL). Tau‐PET SUVRs in entorhinal cortex (ERC) and a temporal MetaROI were z‐scored using tracer‐specific reference cohorts of cognitively normal, Aβ‐ adults aged 60‐70yrs. Key demographic and genetic data (sex, race/ethnicity and APOE genotype) were obtained for each cohort and harmonized. Robust linear models predicting tau burden were used to test for interactions between these variables and continuous Aβ in a pooled sample and in cohort‐specific models. Cohort and age were covariates.

**Result:**

A total of 2,114 cognitively normal older adults were included across 4 cohorts which differed on many characteristics (Table 1). In pooled analyses, there was a sex by Aβ interaction on ERC (but not MetaROI) tau such that women had disproportionately more tau as Aβ increased (Table 2; Figure 1 shows raw data). In models focused on race/ethnicity, non‐Hispanic white individuals had more tau as Aβ increased compared to Black individuals. In models predicting ERC or MetaROI tau, e3/e4 or e4/e4 (versus e3/e3) individuals had higher tau as Aβ increased, respectively. Age and global Aβ were significant predictors in all models. In cohort‐specific analyses, findings were inconsistently replicated such that some results from the pooled sample were not observed in any individual cohort, and some cohort‐specific results were not observed in the pooled sample (Table 2).

**Conclusion:**

In a combined cohort analysis, sex, race/ethnicity and APOE interacted with Aβ on tau burden in unimpaired older adults. However, there was little between‐cohort consistency in associations between sex, race/ethnicity, APOE, and AD biomarkers, suggesting that cohort heterogeneity and statistical power have major influence on the observation of these effects.